# Do Treponemes in Bovine Digital Dermatitis-Associated Claw Horn Lesions Preclude Co-Infection by Pyogenic Bacteria?

**DOI:** 10.3390/microorganisms14071542

**Published:** 2026-07-15

**Authors:** Sabine Brandt, Mariella Zimmermann, Edmund K. Hainisch, Sonja Weigelsperger, Ralf Steinborn, Johann Kofler

**Affiliations:** 1 Research Group Oncology (RGO), Centre for Equine Health and Research, Department for Small Animals and Horses, University of Veterinary Medicine, Veterinaerplatz 1, 1210 Vienna, Austria; edmund.hainisch@vetmeduni.ac.at; 2 Clinical Department for Farm Animals and Food System Science, Clinical Center for Ruminant and Camelid Medicine, University of Veterinary Medicine, Veterinaerplatz 1, 1210 Vienna, Austria; mariella.zimmermann@yahoo.com (M.Z.); johann.kofler@vetmeduni.ac.at (J.K.); 3 VetCore Genomics, University of Veterinary Medicine, Veterinaerplatz 1, 1210 Vienna, Austria; sonja.weigelsperger@vetmeduni.ac.at (S.W.); ralf.steinborn@vetmeduni.ac.at (R.S.)

**Keywords:** cattle, lameness, digital dermatitis, *Treponema* ssp., claw horn lesions, deep digital sepsis, bacterial interference

## Abstract

Bovine digital dermatitis (BDD) is an infectious claw disease chiefly induced by treponemes, while deep digital sepsis (DDS) is caused by pyogenic bacteria. It is currently unclear why persistent BDD-associated claw horn lesions (BDD-CHL) do not develop DDS. One possible explanation could be that the intralesional presence of treponemes makes it more difficult for pyogenic bacteria to colonize BDD-CHL. To lay the groundwork for exploring this possibility, we determined the presence and amounts of treponemes and selected pyogenic bacteria in BDD-CHL versus DDS at the DNA level. “Total Treponema” (TT-)PCR and type-specific qPCR were used to screen 18 BDD-CHLs from 15 cows and 18 DDS samples from 11 cows for bacterial DNA. TT-PCR identified *Treponema* DNA as most abundant DNA in 8/18 BDD-CHLs and 1/18 DDS samples. *qPCR* revealed *Treponema pedis* DNA in all BDD-CHLs, and one DDS lesion. *Fusobacterium necrophorum* DNA was significantly more abundant in DDS compared to BDD-CHL samples (*p* = 0.027). Both lesion types harbored similar mean levels of *Trueperella pyogenes* DNA. Interestingly, 7/18 DDS samples contained *Parvimonas* DNA. Our DNA-based data call for downstream studies involving living BDD-CHL-associated *Treponema* species and *F. necrophorum*, and, importantly, point to a possible pathogenic role of *Parvimonas* in DDS.

## 1. Introduction

Bovine digital dermatitis (BDD) is a common infectious disease characterized by ulcerative epidermal and dermal claw lesions [1,2,3]. Acute cases of BDD cause substantial pain and lameness [4,5] leading to reduced milk yield, lower fertility, and decreased weight gain [4,5,6,7,8]. Thus, BDD not only compromises the welfare of affected animals, but also causes significant economic losses [9,10], all the more since the incidence of disease is increasing worldwide [3,11,12]. In 2022, 55.8% of Austrian dairy herds were shown to be endemically affected by BDD [12]. In some regions of the world, the prevalence of BDD can reach 93% at the herd level [11].

The common form of BDD presents as a cutaneous disease affecting the skin around the claws and/or the interdigital spaces. It can be classified into the stages M0, M1, M2, M3, M4, or M4.1 according to the ‘M-scoring system’ [1,2]. Efforts to elucidate the etiology of disease have led to the identification of *Treponema* species in conjunction with unhygienic stabling as major causative factors [13,14,15,16,17]. In addition, several reports suggest that other anaerobic bacterial genera, notably *Mycoplasma* [18,19,20,21], *Porphyromonas* [21,22], *Fusobacterium* [21,23], and *Dichelobacter* [20,23] may be involved in disease onset and progression. However, these reports are far from providing a consistent picture so that the role of bacteria other than treponemes in BDD remains unclear.

As a different manifestation of common BDD, disease may present as ‘non-healing’ claw horn lesions that specifically occur in endemically BDD-affected herds [24,25,26]. The lesions comprise white line abscesses, sole ulcers, toe ulcers, toe necrosis, and penetrating claw horn fissures with exposed dermis. Alike common BDD lesions, they harbor *Treponema* species such as *T. pedis*, *T. phagedenis*, *T. medium*, *T. denticola*, and *T. refringens* [13,14,15,16,17] that also contaminate the farm environment, including barns, pastures, and walkways [24,25]. Since ‘non-healing’ claw horn lesions with exposed dermis contain the same *Treponema* species as BDD M2-stage lesions [25,27], they are meanwhile termed ‘BDD-associated claw horn lesions’ (BDD-CHL) [27,28].

BDD-CHLs are usually painful and long-lasting, resulting in persistent lameness [25,27,29]. Conventional methods such as the removal of loose perilesional horn and the application of a block are effective in the treatment of common white line abscesses and sole ulcers. However, BDD-CHLs usually fail to respond to this approach when implemented by farmers or hoof trimmers [24,25,26]. Persistent BDD-CHLs thus constitute a serious welfare issue in endemically affected herds and require professional and sustained veterinary management [25,26,27,28].

Initial diagnosis of BDD-CHL is based on the clinical appearance of the lesions. Following hoof trimming and the removal of loose perilesional horn, a characteristic pungent, fetid smell is noted, similar to what is observed in cases of acute M2-stage BDD. Endemic occurrence of BDD in the herd of the affected individual further supports BDD-CHL diagnosis [25,26,28,30].

Macroscopically, BDD-CHLs typically display hypergranulation, and resemble acute BDD lesions (M2 stage) in shape and surface texture [27,28]. Affected bovines commonly exhibit a higher degree of lameness compared to cattle with acute BDD [4,28,31]. BDD-CHLs can persist for several months [25,26,27] or more than a year [28] and involve the posterior wall and sole segments. In such cases, the heels of the affected claws display an unusual height that often corresponds to the length of the dorsal wall (“square feet”) [32].

Deep digital sepsis (DDS) arises from sole ulcers, toe ulcers, white line abscesses [33], interdigital phlegmon [34], or wounds penetrating the distal interphalangeal (DIP) joint or the digital flexor tendon sheath (DFTS) [33,35,36]. The common cause of sepsis is primary or secondary infection by pyogenic bacteria. These pathogens successively invade deeper structures when the primary dermal disease is not treated properly at an early stage [37]. Bacterially infected deeper structures can include the deep flexor tendon at the insertion site, the flexor tubercle of the pedal bone, the distal sesamoid bone, the podotrochlear bursa, the apex of the pedal bone, the distal and/or the proximal interphalangeal joint, and/or the DFTS [33,35,37]. DDS is commonly unilateral and characterized by a moderate to severe, bulge-shaped, circumferential swelling involving the coronet and/or the bulbs of the heel [33,36] (the second figure of Section 2.1).

Pyogenic bacteria causing DDS commonly include *Bacteroides pyogenes*, *Escherichia coli*, *Fusobacterium necrophorum*, and *Streptococcus* as well as *Trueperella* species [38,39].

Surprisingly, long-term monitoring of BDD-CHLs during treatment revealed that deep supporting structures of the affected claw only rarely develop sepsis [28,31] even though the lesions commonly persist for several months [25,26,27]. One explanation for this frequent observation could be that BDD-specific *Treponema* species competitively antagonize pyogenic bacteria, thus preventing them from invading deeper claw structures [40,41,42].

To lay the groundwork for exploring this possibility, we hypothesized that a growth advantage for treponemes over pyogenic bacteria in BDD-CHL is reflected in their respective DNA quantities. To test this hypothesis, we collected 18 BDD-CHL and 18 DDS samples from different affected feet or sites of 15 and 11 dairy cattle, isolated DNA therefrom, and conducted a comparative pilot study at the DNA level. Our first objective was to establish the *Treponema* infection status in BDD-CHL versus DDS using consensus PCR followed by Sanger amplicon sequencing. Our second objective was to determine and comparatively evaluate the intralesional copy numbers of *Treponema pedis* versus selected pyogenic bacteria DNA using qPCR to elucidate whether BDD-CHLs and DDS samples differ with respect to the presence and DNA load of the selected bacteria.

## 2. Materials and Methods

### 2.1. Diagnosis

**Figure 1:**
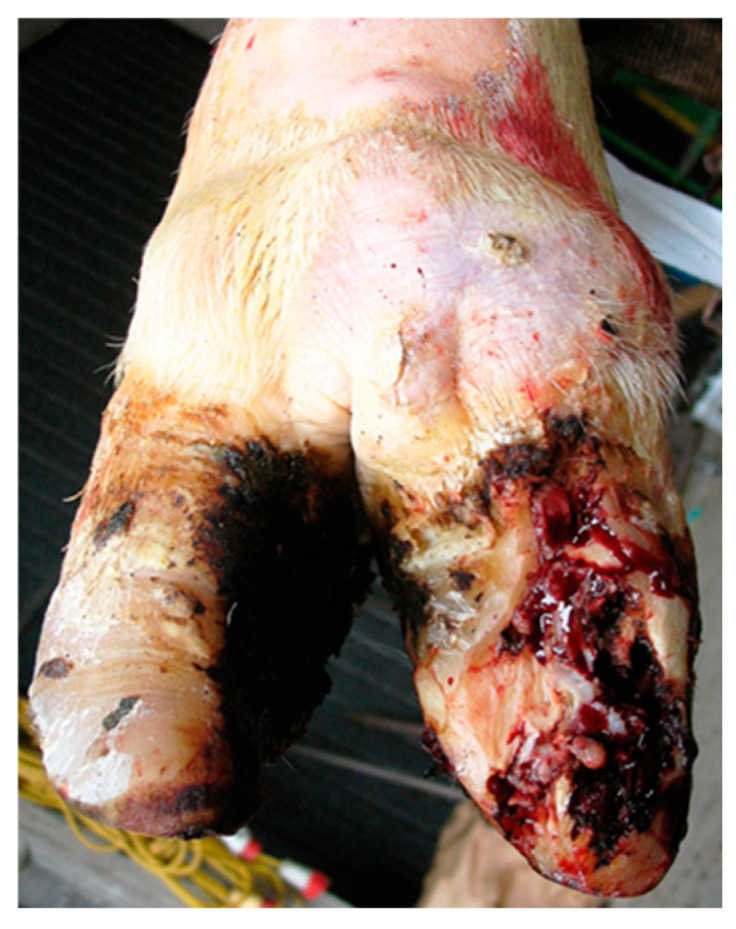
Dorsal view of a BDD-associated dorsal horn fissure. Note the exposed dermis, hypergranulation of the dermis and apparent circumscribed swelling dorsally on the coronet. BDD-CHL is typically associated with a chronic infection of the dermis by *Treponema* species in the abaxial, dorsal, or axial region of the horn wall or the sole. As a result, mild to moderate, firm, and usually painless swelling develops in the coronet area dorsally, abaxially, or on the bulb area of the heel plantarly, depending on the specific location of the BDD-CHL [28].

**Figure 2:**
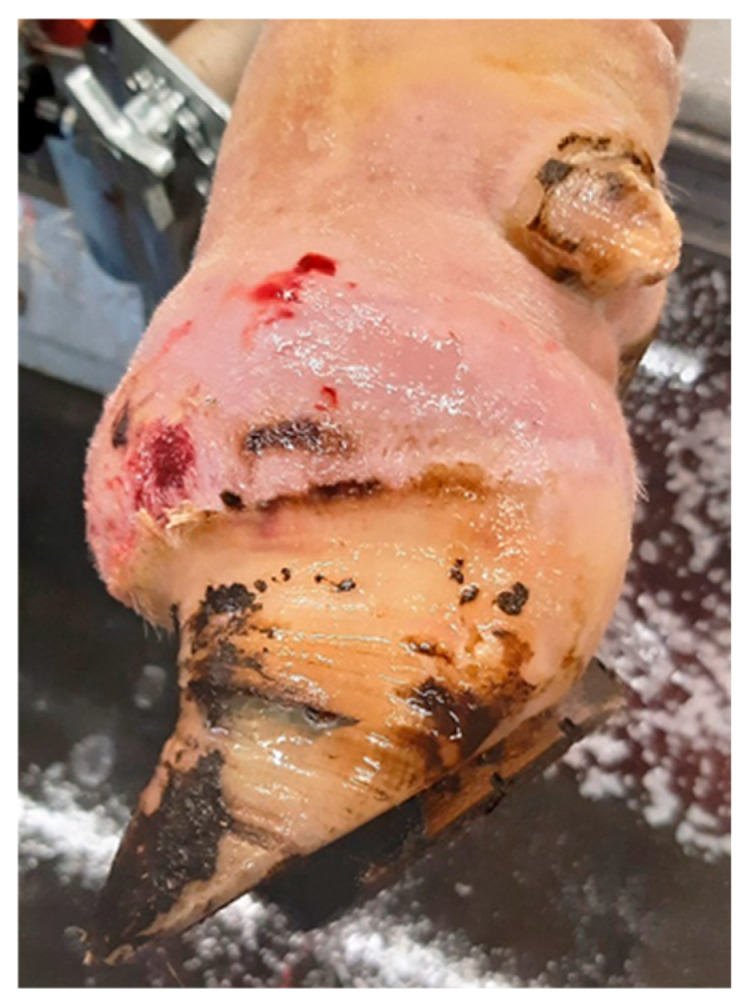
Lateral view of a case of DDS originating from a white line abscess. Note the severe circumferential swelling on the coronet and the bulb of the heel, indicating a purulent arthritis of the distal interphalangeal joint, as well as the moderately hyperextended lateral claw, indicating necrosis of the deep digital flexor tendon at the insertion site.

The diagnosis of BDD-CHL on the sole and wall of affected claws was made according to the characteristic clinical presentation of the disease [24,25,28] (Figure 1). The diagnosis of DDS was based on the presence of a primary claw lesion (sole ulcer, white line abscess, interdigital phlegmon, perforating wound at the coronary band or on the bulb of the heel, and a moderate to severe, bulge-shaped swelling at the bulb of the heel, or circumferentially around the entire coronet and the bulb of the heel (Figure 2). Further diagnostic criteria included the presence of a fistula with purulent discharge that could extend several centimeters towards the distal sesamoid bone, the pedal bone, the DIP joint, and/or the DFTS, and the formation of a hyperextended claw. Ultrasonographic and radiological examination of the affected digit confirmed the presence of deep, mainly unilateral digital sepsis with fibrinopurulent inflammation of the pedal bone, the distal sesamoid bone, and/or the DIP-joint and/or the DFTS [33,34,35,36,43,44].

### 2.2. Sample Collection

In 2023, 18 tissue samples were obtained from 15 cows with BDD-CHL (Figure 1), and another 18 samples were collected from 11 cows with DDS (Figure 2).

All lesions were superficially cleaned with water (from a water hose) and subsequently rinsed with 0.9% saline solution prior to therapeutic surgery, during which the samples were taken. In cases of BDD-CHL, the tissue samples were collected from different affected feed during wound debridement. In cases of DDS, treatment included the resection of the distal sesamoid bone and the deep flexor tendon at its insertion site, digital amputations, and the resection of the flexor tendons at one of the two DFTS. DDS samples were collected from different feet of affected cows but also from different sites of one affected foot in some cases (e.g., samples 9a–c) as shown in Table 1. The samples were taken from primary dermal lesions on the claw or from primary skin wounds such as white line disease (WLD), sole ulcers, or infected wounds, but not from secondarily infected deep supporting structures.

All surgical interventions were conducted following the administration of intravenous regional anesthesia [31,35,36]. Samples were placed in a sterile plastic tube, immediately frozen and stored at −20 °C until DNA extraction. Sample details are shown in Table 1. Supplementary Table S1 provides details such as breed, age, gender, and locomotion score of the animals from which the samples were collected.

### 2.3. DNA Extraction and Validation

DNA was extracted from all samples using a DNeasy Blood & Tissue Kit (Qiagen GmbH, Hilden, Germany) according to the instructions of the manufacturer. The DNA yields were determined using a NanoDrop spectrophotometer (Implen, Munich, Germany). The PCR-compatible quality of DNA isolates was assessed by PCR for the house-keeping gene β-actin that is present in every eukaryotic cell [45]. In case the reaction yielded a negative result, the entire procedure starting from DNA isolation was repeated.

### 2.4. Qualitative PCR Screening for Treponema DNA and Sequence Analysis

In a next step, DNA isolates were screened for the presence of *Treponema* DNA by “Total Treponemal PCR” (TT-PCR) according to Moe et al. (2010) [46]. The “universal” TT PCR primers bind to a region that is conserved among treponemes and some other genetically closely related bacteria [47]. The 20 µL reaction mixtures consisted of 1× HF buffer (ThermoFisher Scientific, Vienna, Austria), 0.6 µL DMSO (100%; Thermo Fisher Scientific), 0.4 µL 10 mM of each dNTP (ThermoFisher Scientific), 0.5 µL of each primer (100 pmol/µL; Eurofins, Vienna, Austria), 0.5 units of Phusion™ Hot Start II DNA Polymerase (Thermo Fisher Scientific) and 1 µL of template DNA.

Amplification reactions were conducted in a Life Eco Thermocycler (Biozym, Hessisch-Oldendorf, Germany) and included a positive control (*Treponema*-positive BDD-DNA), a negative control (healthy claw skin DNA), and a no-template control (sterile water). Denaturation at 98 °C for 2 min was followed by seven touchdown cycles (98 °C for 15 s, 72 °C to 69 C for 30 s [−0.5 °C per cycle], 72 °C for 30 s) and then 40 cycles at 98 °C for 15 s, 69 °C for 30 s, and 72 °C for 30 s. The final elongation step was performed at 72 °C for 5 min.

PCR products (16 µL aliquots) were separated by gel electrophoresis on a 1.5% TAE agarose gel and visualized by ethidium bromide staining. Amplicons of the expected size (657 bp) were excised, purified using a GeneJET Gel Extraction Kit (Thermo Fisher Scientific) according to the instructions of the manufacturer, and then subjected to bidirectional Sanger sequencing (Eurofins).

The resulting 5’ and 3’ sequences were aligned pairwise to each other and then to the bacterial DNA sequences available in the GenBank using the Basic Local Alignment Search Tool (BLAST Version 2026) (https://blast.ncbi.nlm.nih.gov/Blast.cgi, accessed on 15 July 2025).

### 2.5. Quantitative Detection of Pyogenic Bacteria and Treponema pedis DNA by qPCR

To address intralesional DNA levels of pyogenic bacteria and *T. pedis*, qPCR from all but one DNA aliquots (5b; exhausted) was carried out in 15 µL volumes, containing 250 nM of each primer, 0.4 × EvaGreen dye (Biotium, Fremont, CA, USA) or 200 nM of the hydrolysis probe, 1 × PCR buffer B2 (Tris-HCl, (NH_4_)_2_SO_4_ and Tween^®^ 20; Solis Biodyne, Tartu, Estonia), 3.5 mM MgCl_2_, 200 nM of each dNTP (Solis Biodyne), 1 unit of hot-start Taq DNA polymerase (HOT FIRE Pol^®^; Solis Biodyne) and 2 µL (40 ng) of template DNA. All primer and probe sequences are specified in Table 2.

The specificity of the qPCR assays was evaluated in silico using NCBI’s Primer-BLAST (https://www.ncbi.nlm.nih.gov/tools/primer-blast/; accessed on 7 March 2026). Oligonucleotides were checked for matches in two DNA sequence databases: the general NCBI nucleotide database limited to bacterial sequences, and the RefSeq set of representative genomes.

DNA samples were assayed in duplicates along with positive control DNA isolated from the respective target species and a no-template control (NTC; sterile water). Cycling conditions consisted of an initial preincubation step at 95 °C for 15 min followed by 40 amplification cycles of denaturation at 95 °C for 15 s, combined annealing and elongation at 60 °C for 1 min in case of probe-based monitoring, or separate annealing at 60 °C for 25 s, and elongation at 72 °C for 30 s in case of dye-based qPCR-melting curve analysis (qPCR-MCA). For the MCA step, qPCR products were gradually heated from 65 to 93 °C and the fluorescence signal was consistently acquired during this process to identify the exact temperature where the qPCR product denatures. The 96-well qPCR was run on the qTOWER^3^ (Analytik Jena, Jena, Germany). Raw fluorescence data were exported from the “Raw Data” tab of the LightCycler 96 software version 1.1.0.1320 or as “.csv” file from the “Monitoring” submenu of the qTOWER^3^ software version 4.1.3.0. qPCR efficiency was determined in silico by fitting a regression line to the log-linear phase of non-baseline-corrected fluorescence data using the Real-time PCR Miner software Version 4.0 [48] (http://miner.ewindup.cn). Median efficiency was determined from all positive replicates of the assays (Table 2).

“Adjusted” quantification cycle (Cq) values referring to a 100% exponential-phase efficiency (E) were calculated using the formula Cq_adj_ = Cq × log10(E+1)/log10(2), where Cq represents the “raw” Cq value and E the efficiency of the reaction [49]. Absolute target copy numbers (N) per 2 µL of template DNA were subsequently calculated from adjusted Cq values using the equation: N = 10 × 2^(35-Cq)^ based on the recommendations by Ruiz-Villalba et al. [50]. Accordingly, samples with Cq values > 35 were considered as qPCR-negative.

Calculated copy numbers per 2 µL of sample were translated into bar charts (Section 3.3) for their presentation. The (mean) copy numbers/2 µL per cow were used to calculate median values and interquartile range (IQR) values per group and bacterial species using a free online IQR calculator (https://www.socscistatistics.com/tests/iqr/calculator/, accessed on 15 July 2025) (see table qPCR-positive samples: numerical summary).

### 2.6. Statistics

The target variable was the species-specific bacterial DNA load (DNA/µL) calculated from efficiency-corrected Cq values. The distribution of the target variable was not normal in any group, as determined by a Shapiro–Wilk test (https://www.statskingdom.com/Shapiro–Wilk-test-calculator.html, accessed on 4 July 2026) (the significance level α was uniformly set at 0.05). Consequently, the Mann–Whitney U test was used to determine whether BDD-CHL- and DDS-derived samples significantly differed with respect to the species-specific bacterial DNA loads (https://www.statskingdom.com/170median_mann_whitney.html, accessed on 4 July 2026). Given that several samples derived from one or different claws of the same animal, the test was applied at the animal level using mean bacterial DNA loads per affected cow. In case the data contained ties, i.e., identical values in different individuals, the Mann–Whitney U test was carried out with the normal approximation method that uses the ties correction, instead of the exact calculation method. A Bonferroni correction was applied because three hypotheses were tested. To this aim, the significance level α was divided by the number of hypotheses, resulting in a Bonferroni-corrected alpha level of α = 0.05/3 = 0.016.

## 3. Results

### 3.1. Bacterial DNA Identified by TT PCR in BDD-Associated Claw Horn Lesions

**Figure 3:**
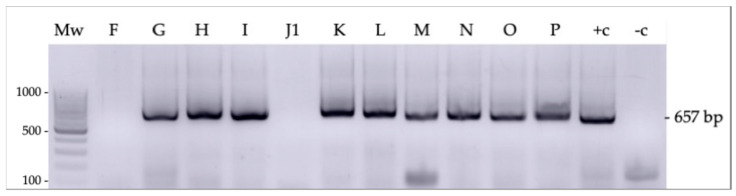
TT PCR from BDD-CHL DNA isolates. The exemplary gel electrophoresis image shows amplification products of expected size (657 bp) obtained from 9/11 samples. As depicted, isolates F and J1 scored negative. This finding agreed with negative β-actin PCR. Extraction of new DNA from F and J1 solved the problem. Mw: molecular weight marker (ten DNA fragments with a size range of 100–1000 bp; 100 bp DNA ladder; ThermoFisher Scientifics); +c: positive control, i.e., *T. pedis*-positive BDD DNA; −c: negative control (normal bovine skin DNA).

The 18 BDD-CHL DNA isolates were first subjected to β-Actin PCR that scored positive in 16/18 cases. DNA isolates F and J tested negative. Subsequent TT PCR yielded amplification products of expected size from all β-Actin PCR-positive DNA isolates (Figure 3). Newly extracted F and J1 DNA scored positive by β-Actin and TT PCR.

In 8/18 BDD-CHLs from 8/15 cows, amplicon sequencing identified *Treponema* DNA as the most abundant TT PCR target. The other samples contained *Bacteroidia* DNA first isolated from the feline oral cavity (5/18 samples), DNA from uncultured bacteria previously detected from ovine foot rot (2/18 samples; unpublished), or DNA of yet uncharacterized bacteria first isolated from the human oral cavity (1/18 samples) or BDD (2/18 samples) as most abundant target molecules (Table 3).

### 3.2. Bacterial DNA Identified by TT PCR in DDS Lesions

TT PCR scored negative for 6/18 DDS samples originating from five cows. In 7/18 samples (five animals) *Parvimonas* DNA was detected as most abundant target. A *Fusobacterium mortiferum*-like sequence genetically 98% identical to *F. necrophorum,* and uncharacterized *Bacteroidetes* DNA were each detected in one DNA isolate. One DDS lesion contained a bacterial sequence that was previously detected by us from ovine foot rot (MW726636; unpublished). In case of the amplicon obtained from sample #1, bidirectional sequencing was repeatedly unsuccessful (Table 4).

The DNA sequences of the bacteria detected by TT PCR have been submitted to GenBank and obtained the accession numbers PZ427686-PZ427707.

### 3.3. qPCR Results

**Figure 4:**
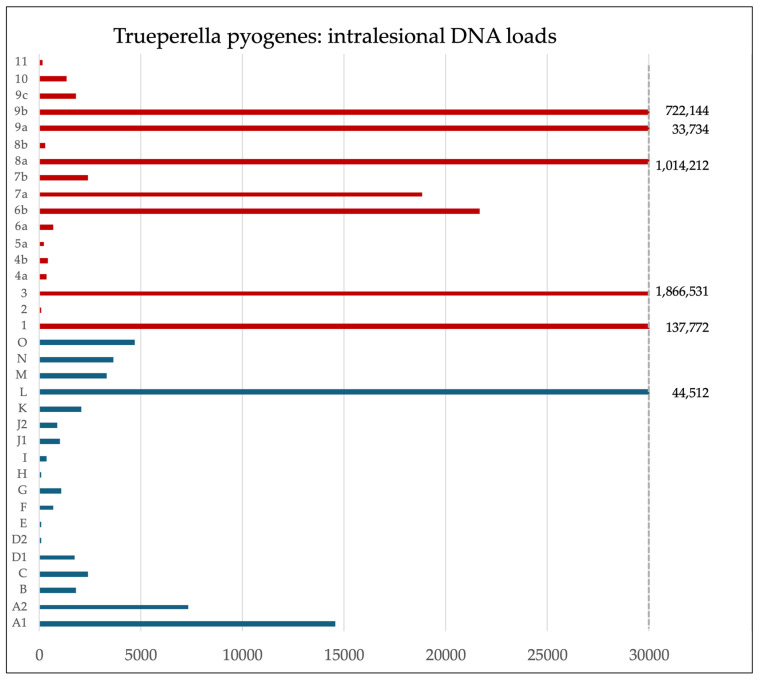
Intralesional copy numbers of *Trueperella pyogenes* DNA. *T. pyogenes* DNA loads in DDS are shown in red, and in BDD-CHL in blue. Given their wide range, loads exceeding 30,000 copies per 2 µL are specified numerically in the figure.

**Figure 5:**
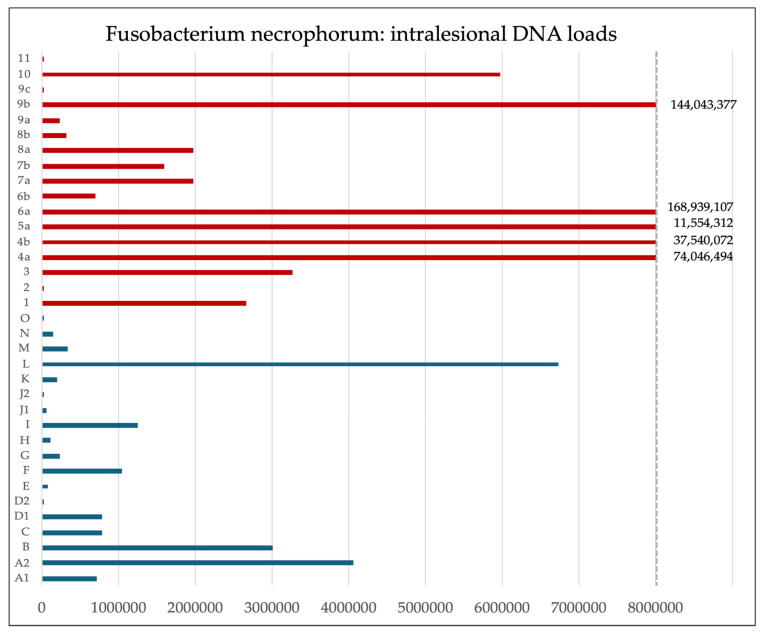
Intralesional copy numbers of *Fusobacterium necrophorum* DNA. *F. necrophorum* DNA loads in DDS are shown in red, and in BDD-CHL in blue. Given their wide range, loads exceeding 8 × 10^6^ copies per 2 µL are provided numerically in the figure.

Quantitative PCR detection of selected pyogenic bacteria and of *Treponema pedis* as major causative agent of BDD was carried out from 18 BDD-CHL and 17 DDS DNA isolates. Regardless of their origin, all DNA samples harbored measurable amounts of *Trueperella pyogenes*, and *Fusobacterium necrophorum* (Figure 4 and Figure 5), but not *Escherichia coli* according to *ybbW* qPCR-melt curve analysis [51]: qPCR products obtained from five samples across both disease groups (BDD-CHL: #B, #J, #P; DDS: #4, #6) melted at a higher temperature (88.1 °C) than the *E. coli* control (83.7 °C), pointing to the amplification of an alternative target, i.e., *Escherichia whittamii*, *Shigella boydii,* or *Shigella dysenteriae* (Table 2).

Copy numbers of *T. pyogenes* DNA calculated from Cq values did not significantly differ between BDD-CHL- and DDS-derived samples as reflected by U = 63 (*p* = 0.33). In contrast, the calculated *F. necrophorum* DNA levels per animal were significantly higher in DDS compared to BDD-CHL (U = 87.00, *p* = 0.027). When applying the Bonferroni correction, this difference was not significant.

**Figure 6:**
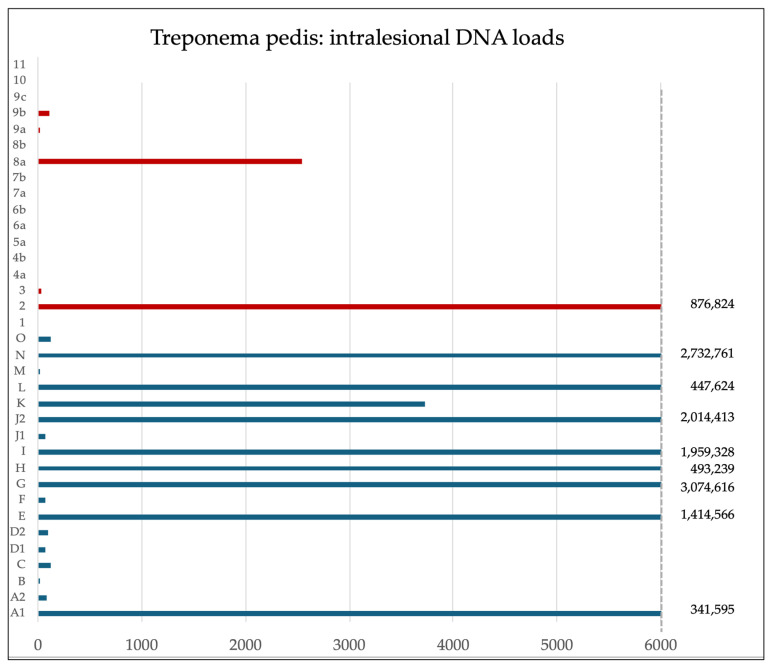
Intralesional copy numbers of *Treponema pedis* DNA. *T. pedis* DNA loads in DDS are shown in red, and in BDD-CHL in blue. Given their wide range, loads exceeding 6000 copies per 2 µL are specified numerically in the figure.

Variable amounts of *Treponema pedis* ranging from <10 to >3 × 10^6^ DNA copies per 2 µL were detected in 100% of BDD-CHL-derived DNA isolates but in only 5/17 DDS samples obtained from four animals (Figure 6). The two sample groups showed a highly significant difference regarding the intralesional abundance of *T. pedis* DNA (U = 146, *p* = 0.0009599).

*Dichelobacter nodosus* DNA was solely detected in sample B (20 copies per 2 µL DNA template). None of the samples contained detectable amounts of *Staphylococcus aureus* DNA.

Table 5 provides a numerical summary of data for the lesions/animals that scored positive for *T. pyogenes*, *F. necrophorum*, and *T. pedis* qPCR.

## 4. Discussion

In BDD-associated claw horn lesions (BDD-CHL), BDD-specific *Treponema* species colonize the superficial and the deeper layers of the epidermis as well as the dermis. However, histological studies on acute M2-lesions and the histopathological and molecular biological characterization of BDD-CHLs have shown that BDD-specific treponemes do not invade subcutaneous tissue, muscles, or bones [1,17,27,52].

In stark contrast, BDD-unrelated lesions such as sole ulcers or white line abscesses are commonly associated with purulent infections of the deeper supporting structures of the claw [33,36]. This complication termed DDS notably develops when the primary disease of the sole or claw wall is not treated promptly and accurately [33,37].

A common feature of BDD-CHL and DDS is a moderate to severe circumscribed swelling [33,36] that can point to an advanced infection of the distal interphalangeal joint. Yet, swelling can also occur when the purulent infection has destroyed the deep digital flexor tendon at the insertion site, resulting in an overextended claw [33,36]. In long-lasting BDD-CHL, prominent circumscribed swelling of the coronet and/or the bulb of the heel [28,33,36] may be therefore misinterpreted as having reached the pedal bone or distal interphalangeal joint and entail the unnecessary amputation of the affected claw [25]. Meanwhile, there is robust evidence that careful surgical wound debridement by removal of the infected dermal tissue under local anesthesia can achieve complete healing of BDD-CHLs within two to approximately eight weeks without the need for claw amputation [28,31,53,54]. Consequently, diagnostic tools allowing the clear distinction between BDD-CHL and DDS are essential for correct therapeutic decisions. In this context, the identification of disease-specific bacteria and investigations on their interplay can help refining BDD-CHL and DDS diagnosis by molecular biological approaches such as qualitative PCR, qPCR, or digital PCR [55].

BDD-CHLs often persist for several months to more than a year. On these grounds, one would expect to detect DDS in BDD-CHL, especially when disease is long-lasting. Yet, the infection of the exposed dermis by BDD-treponemes in cases of CHLs rather seems to prevent the development of DDS [28,31]. There is currently no explanation for this phenomenon. A biological mechanism termed ‘bacterial interference’ may protect *Treponema*-associated lesions from infection by pyogenic bacteria similar to what is reported for other bacterial species [40,41,42]. Bacterial interference is a well-known phenomenon described as antagonism between bacterial species during the process of surface colonization and nutrient acquisition [42]. It also confers microorganisms with the ability to protect their host organisms from invading microbial pathogens by impairing their adhesion and toxic effects [56]. The concept of passive and active bacterial interference is widely used in human medicine [40,57,58]. It consists of using low-virulence bacteria to competitively prevent infections by pathogenic bacteria [41].

The herein presented study was conducted using ex vivo material collected from BDD-CHLs and DDS lesions in the course of treatment. The types of lesions were anatomically, morphologically, and pathologically heterogenous and so were the samples collected therefrom. This heterogeneity represents a strength of the study in that it provides general insights, but also a limitation as it may influence results. This has to be kept in mind when interpreting obtained data. The study aimed at investigating whether BDD-CHL significantly differ from DDS with respect to the colonization by selected pyogenic bacteria at the DNA level. The idea underlying this approach was to obtain initial indications of a possible interference between typical pyogenic bacteria and treponemes in persistent BDD-CHLs without deep sepsis. It is currently assumed that BDD-CHL are co-induced by BDD-associated treponemes [27,28]. Hence, BDD-CHL and DDS DNA isolates were first assessed for the presence of treponemal DNA to confirm that *Treponema* ssp. preferentially occur in BDD-CHL. Once the most abundant *Treponema* ssp. DNA and the *T. pedis* DNA loads in the lesions were determined, we assessed whether BDD-CHLs would contain significantly lower copy numbers of pyogenic bacteria DNA when compared to DDS. Treponemal DNA was predominantly detected in tissue samples from cattle with BDD-CHL as the most abundant target DNA. The identified *Treponema* species included *Treponema pedis, T. denticola, T. phagedenis,* or *Treponema* clone PT1 as previously reported for BDD [15,16,25]. A recently published study also found *T. pedis* DNA to prevail in BDD-CHLs in Switzerland [27]. Only one DDS was scored positive by TT PCR and harbored *Treponema DNA* as most abundant target DNA. This finding was confirmed by qPCR that revealed high *T. pedis* copy numbers in this sample. In several BDD-CHL samples, TT PCR followed by amplicon sequencing led to the identification of feline oral *Bacteroidia*. This taxon was initially isolated from the oral cavity of a cat and may have an active role in periodontal disease [59]. This finding, and the presence of uncharacterized bacteria isolated from the human oral cavity or ovine foot rot in BDD-CHL samples underscore the significant overlap between bacterial pathogens detected from BDD, ovine DD, and equine hoof canker on one hand, and periodontitis in mammal species including humans on the other [47,60,61,62,63].

Interestingly, more than half of the DDSs contained *Parvimonas* DNA as most abundant target DNA. This finding was surprising because there is no published evidence on the occurrence of *Parvimonas* ssp. in bovine claw lesions so far. Given the high sequence homology, the detected *Parvimonas* sequences likely represent *P. micra*–anaerobic bacteria that present as small, non-spore-forming, Gram-positive, anaerobic cocci [64]. Originally known as *Peptostreptococcus micros* [29], *P. micra* frequently colonize the oral cavity, where they are associated with periodontal disease [65]. The pathogen is also involved in joint inflammation in prosthetics, diabetic foot infections, and liver and spleen abscesses in humans [29]. Recent evidence further indicates that *P. micra* promotes metastasis in human oral squamous cell carcinoma by activating the transcriptional activator hypoxia-inducible factor 1-alpha and autophagy signaling via the CKAP4-RanBP2 and -NBR1 pathways [66]. Our findings suggest the possibility that *P. micra* or closely related bacteria may also have a pathogenic role in DDS. This interesting possibility will be subject to in-depth investigations in the near future.

In the UK, *F. necrophorum* was frequently detected in BDD-associated white line abscesses, sole ulcers, and toe necrosis [67]. These data differ from results presented by Alsaaod and colleagues [27], yet agree with herein presented findings, i.e., the consistent detection of substantial amounts of *F. necrophorum* DNA from both types of lesions. However, BDD-CHL and DDS statistically differed with respect to *F. necrophorum* DNA loads, with DDS harboring significantly higher amounts of *F. necrophorum,* than BDD-CHLs. When applying the Bonferroni correction, this difference is not significant. However, application of this correction is overly conservative as the bacterial species occurring in the lesions likely correlated. Given that the study was exclusively conducted on DNA level, it would be too speculative to deduce an active contribution of treponemes to this finding. However, obtained qPCR data suggest that BDD-CHLs may not provide ideal growth conditions for *F. necrophorum* when compared to DDS. This possibility merits to be further investigated in the future.

*Trueperella pyogenes* DNA loads did not significantly differ between BDD-CHL and DDS. This finding was somewhat surprising since none of the BDD-CHLs displayed signs of deep sepsis. On the other hand, intralesional detection of *T. pyogenes* DNA does not provide any information on the activity of the pathogen.

Interestingly, data from Iowa, USA [20], and especially Brazil [23] point to a causative co-involvement of *Dichelobacter nodosus* in BDD. We detected *D. nodosus* DNA only in a single BDD-CHL lesion at low copy numbers. Discrepancy of data may be explained by differences regarding the animals enrolled, the husbandry conditions, and the health management. In addition, it is unclear to what extent BDD lesions can reflect BDD-CHL with respect to bacterial infection and composition. Dias et al. [30] demonstrated that BDD-associated *Treponema* species were exclusively detected in BDD-affected lesions, BDD-foot and other skin lesions, as well as in healthy skin of cows in BDD-affected herds. In contrast, BDD-unspecific treponemes were found in samples from cows in both, BDD-negative and -affected herds [30]. These results may support the assumption that all CHL with exposed corium in cows from dairy farms with an endemic BDD status are infected by BDD-specific treponemes. Even within BDD lesions, the microbiota is subject to changes that are specific to the morphological stage of BDD development [20]. While early-stage lesions contain numerous bacterial taxa but *Treponema* ssp. in relatively low abundance, advanced lesions are characterized by high amounts of treponemes at the expense of other bacterial species. Moreover, a stage-specific shift in the composition of *Treponema* species has been reported for BDD. Whereas early lesions were shown to harbor mainly treponemes identified as PT1, PT2, PT3, and *T. refringens*-like, advanced lesions predominantly contained *T. pedis, T. medium, T. denticola* and clone PT8. Only *T. phagedenis* was present at all stages of disease [17,20]. In the present study, we detected *T. pedis* DNA by qualitative TT PCR as the most abundant bacterial DNA in three BDD-CHL, and by qPCR in all BDD-CHL at varying levels. Provided that the suggested correlation of *T. pedis* infection with advanced BDD [20] can also be established for BDD-CHL, *T. pedis* qPCR would represent an interesting approach for refined disease staging. No *E. coli* DNA was detected from any of the samples irrespective of their origin. Given the high sensitivity of the qPCR approach on one hand, and the omnipresence of *E. coli* in feces on the other, the data shows that the implemented sampling protocol was effective in preventing contamination.

Finally, we note that the detection limit of the qPCR assays was not evaluated in the current study because its validation would be technically challenging, also requiring an assessment of the recovery efficiency of target molecules throughout the nucleic acid extraction process. With respect to nucleic acid extraction, BDD-CHL and DDS samples should be considered as a complex matrix comparable to food or feces. The efficiency of DNA recovery from those complex matrices is usually around 30% or lower, and neglecting this parameter would lead to underestimation of the true number of target microorganisms in the original sample [68].

## 5. Conclusions

Herein presented data provide further evidence of the association of *Treponema* ssp. with BDD-CHL, and first insights on the presence and DNA loads of selected pyogenic bacteria in this type of claw disease. At the DNA level, our findings do not support the theory that treponemes preclude co-infection by selected pyogenic bacteria in the lesions. Yet, they suggest that BDD-CHLs may not constitute an ideal environment for *Fusobacterium necrophorum*. No inhibition of *Trueperella pyogenes* was observed at DNA level. Obtained qPCR data indicate that *Dichelobacter nodosus,* and *Staphylococcus aureus* only rarely occur in BDD-CHL.

The study has several limitations. First, it was carried out at the DNA level, and thus, it does not allow conclusions on the bacterial viability, activity, or functional interference. Ex vivo analyses at the mRNA and protein level, and co-cultivation studies involving BDD-specific *Treponema* ssp. and characteristic pyogenic bacteria will help gain more insights regarding this issue. Other limitations of the study are the heterogeneity of the collected BDD-CHL and DDS samples including sampling from the same animal in some cases, and the relatively small sample size.

To our knowledge, we are the first to identify *Parvimonas micra*-like bacterial DNA in DDS. Given the active pathogenic role of *P. micra* in various human diseases, in depth studies on the exact identity of these bacteria and their possible causative association with DDS in ungulates are in preparation.

## Figures and Tables

**Table 1:** Samples collected from BDD-CHL- and DDS-affected claws.

Sample	Diagnosis	Location of the Lesion	Sample From
BDD-CHL samples (*n* = 18; from different affected feet of 15 cows)			
A1	BDD-associated axial horn fissure	Lateral claw of right hindlimb	lesional tissue
A2	BDD-associated sole ulcer	Lateral claw of left hindlimb	lesional tissue
B	BDD-associated white-line-abscess	Lateral claw of right hindlimb	lesional tissue
C	BDD-associated sole ulcer	Lateral claw of left hindlimb	lesional tissue
D1	BDD-associated white-line-abscess	Lateral claw of left hindlimb	lesional tissue
D2	BDD-associated sole ulcer	Lateral claw of right hindlimb	lesional tissue
E	BDD-associated axial horn fissure and toe ulcer	Lateral claw of right hindlimb	lesional tissue
F	BDD-associated sole ulcer	Lateral claw of left hindlimb	lesional tissue
G	BDD-associated horn fissure	Medial dew claw of right hindlimb	lesional tissue
H	BDD-associated white-line-abscess	Medial claw of left forelimb	lesional tissue
I	BDD-associated axial horn fissure	Lateral claw of right hindlimb	lesional tissue
J1	BDD-associated sole ulcer	Lateral claw of left hindlimb	lesional tissue
J2	BDD-associated axial horn fissure	Lateral claw of right hindlimb	lesional tissue
K	BDD-associated axial horn fissure	Lateral claw of right hindlimb	lesional tissue
L	BDD-associated toe ulcer	Lateral claw of right hindlimb	lesional tissue
M	BDD-associated white-line-abscess	Lateral claw of right hindlimb	lesional tissue
N	BDD-associated axial horn fissure	Lateral claw of right hindlimb	lesional tissue
O	BDD-associated toe ulcer	Lateral claw of right hindlimb	lesional tissue
DDS samples (*n* = 18; from different affected feet or several digital sites per foot of 11 cows)			
1	WLA and purulent arthritis of DIP joint	Lateral claw of left hindlimb	lesional tissue
2	Postsurgical bone infection with sequestrum formation of the pedal bone	Lateral claw of right hindlimb	lesional tissue
3	WLA and purulent inflammation of the distal sesamoid bone and of DDFT at its insertion	Lateral claw of right hindlimb	lesional tissue
4a	Sole ulcer and purulent arthritis of DIP joint with retroarticular abscess	Lateral claw of left hindlimb	retroarticular abscess
4b		Lateral claw of left hindlimb	DIP joint
5a	Sole ulcer and purulent arthritis of DIP joint	Lateral claw of right hindlimb	lateral DIP joint
5b		Medial claw of right hindlimb	medial DIP joint
6a	Sole ulcer and purulent arthritis of DIP joint	Lateral claw of right hindlimb	purulent fistula exudate
6b		Medial claw of left hindlimb	purulent fistula exudate
7a	WLA and purulent arthritis of DIP joint	Medial claw of left hindlimb	purulent WLA fistula exudate
7b		Medial claw of left hindlimb	purulent DIP joint exudate
8a	WLA and fibrino-purulent arthritis of DIP joint; fibrino-purulent tenosynovitis of DFTS	Lateral digit of right hindlimb	purulent DIP joint exudate
8b		Lateral digit of right hindlimb	lateral DFTS exudate
9a	WLA and purulent inflammation of DDFT, distal sesamoid bone, and flexor tubercle; septic serous arthritis of DIP joint	Lateral claw right hindlimb	DDFT exudate
9b		Lateral claw right hindlimb	distal sesamoid bone
9c		Lateral claw right hindlimb	WLA
10	Old, infected cut wound at the dorsal coronet and purulent arthritis of DIP joint	Lateral claw right hindlimb	lesional tissue
11	Sole ulcer and fibrino-purulent arthritis of DIP joint	Lateral claw of left hindlimb	DIP joint exudate

WLA: white-line abscess; DIP joint: distal interphalangeal joint; DDFT: deep digital flexor tendon; DFTS: digital flexor tendon sheath.

**Table 2:** qPCR primers and probes.

Bacterial Species	Target Gene	Reference GenBank ID	Forward (f) and Reverse (r) Primers, and Probes	Product Size (bp)	E (%)
*Dichelobacter* *nodosus* strains B2 &V2	Hydrolase AprB2 (*aprB2*)	B2 CG site: CP144580, FN674446 V2 TA site: CP144658, NC_009446, L38395	DnB2/V2-f: GCAATAGCCAAATTTCTTTAGATGGT DnB2cg-r: CTTTGCGTGGATCAGGACG DnV2at-r: TTTCTTTGCGTGGATCAGGATA DnB2/V2-FAM/BHQ1: TCGCGATGCTGATCCTTTTGACGA	111	86
*Escherichia coli*, *E. whittamii* *Shigella boydii* *S. dysenteriae*	Allantoin Transporter (*ybbW*)	NC_000913.3 NZ_JACSQI010000007 NZ_NIYS01000085 NZ_CP055055	Ecoli_ybbW401-f: TGATTGGCAAAATCTGGCCG Ecoli_ybbW611-r: GAAATCGCCCAAATCGCCAT	211	99
*Fusobacterium* *necrophorum* ssp. *necrophorum*	Autotransporter-associated protein with N-terminal domain (*EO219_08995*)	AF529887 CP034842	Fuson-f: CAGGACTTAAAAATGCAGGAACAA Fuson-r: CTGCTTGTTATATCTGCTGTATTTTCAA	121	94
*Staphylococcus* *aureus*, *Frateuria* *defendens*	Glutamat-synthase with Flavin mono-nucleotide binding domain (*FMN-bgsfp*)	CP047784 NC_007795 NZ_LFQR01000317	S_aureus-f: ACGTTGCATCGGAAACATTG S_aureus-r: TCTCGTATGACCAGCTTCGGTA	81	92
*Treponema* *pedis*	DNA gyrase B (*gyrB*)	CP004120 CP045670 CP061839	TPgyrB-f: GGGCAGGGTCCATTGTAGTT TPgyrB-r: CGCAGTTCAGCGGTACAAG TPgyrB-FAM/ZEN: CCGTCCATTTCGCCCAAACC	74	96
*Trueperella* *pyogenes*	Pyolysin (*plo*)	CP123404 NZ_CP007519	Tp-plo-f: CTCAACGTGGACTTCGATGC Tp-plo-r: CCGAAAACGCTATGTGGAGA	119	100

FAM: 6-Fluorescein; BHQ1: Iowa Black Fluorescent Quencher; ZEN: dark internal quencher (Integrated DNA Technologies, Leuven, Belgium).

**Table 3:** Most abundant bacterial DNA detected by TT PCR from BDD-CHL samples.

Sample	PCR		Most Abundant Bacterial DNA Amplified by TT PCR: Best BLAST Match, Isolation Source	GenBank ID	Maximum Identity
	β-Act	TT			
A1	+	+	Uncultured bacterium clone 071041_137, human oral cavity	JQ468350	95%
A2	+	+	Uncultured bacterium clone 615d, ovine foot rot	MW726636	100%
B	+	+	*Bacteroidia bacterium* feline oral taxon 312, feline oral cavity	KM462160	89%
C	+	+	*Treponema* sp. PT1, BDD	AM942445	97%
D1	+	+	*Bacteroidia bacterium* feline oral taxon 312, feline oral cavity	KM462160	95%
D2	+	+	Uncultured *Bacteroidetes bacterium* clone P-02, BDD	GQ424167	98%
E	+	+	*Treponema pedis* strain DD3F, BDD	KR025849	99%
F	+	+	*Bacteroidia bacterium* feline oral taxon 312, feline oral cavity	KM462160	89%
G	+	+	*Bacteroidia bacterium* feline oral taxon 312, feline oral cavity	KM462160	98%
H	+	+	*Treponema pedis* strain G9JD, BDD	KJ206531	98%
I	+	+	*Treponema pedis* strain DD3F, BDD	KR025849	99%
J1	+	+	Uncultured bacterium clone 615d, ovine foot rot	MW726636	94%
J2	+	+	*Treponema denticola* strain US-Trep chromosome, BDD	CP076573	99%
K	+	+	Uncultured *Treponema* sp. clone 10B549, human oral cavity	FJ976308	99%
L	+	+	*Treponema* sp. clone PT7, *T. denticola*-like, BDD	AM942451	96%
M	+	+	*Treponema phagedenis* subsp. *vaccae* strain g2ST24, BDD	KP063169	99%
N	+	+	Uncultured *Bacteroidetes bacterium* clone P-02, BDD	GQ424167	100%
O	+	+	*Bacteroidia bacterium* feline oral taxon 312, feline oral cavity	KM462160	94%

β-Act: Beta-Actin; TT PCR: “Total Treponemal” PCR.

**Table 4:** Most abundant bacterial DNA detected by TT PCR from DDS samples.

Sample	PCR		Most Abundant Bacterial DNA Amplified by TT PCR: Best BLAST Match, Isolation Source	GenBank ID	Maximum Identity
	β-Act	TT			
1	+	+	No sequence received		
2	+	+	Uncultured *Treponema* sp. clone EQ2_047, equine oral cavity	KT895046	98%
3	+	+	Uncultured *Parvimonas* sp. clone SM10-68, bovine uterus	JN167637	99%
4a	+	−	-		
4b	+	−	-		
5a	+	+	*Fusobacterium mortiferum* strain NCIMB	NR_119089	94%
5b	+	+	Uncultured bacterium clone 615d, ovine foot rot	MW726636	98%
6a	+	+	*Parvimonas* sp. strain PS0692, *P. micra*-like	PQ865214	99%
6b	+	+	*Parvimonas* sp. strain PS0692, *P. micra*-like	PQ865214	98%
7a	+	+	Uncultured *Parvimonas* sp. clone SM10-68, bovine uterus	JN167637	99%
7b	+	+	*Parvimonas* sp. strain PS0692, *P. micra*-like	PQ865214	99%
8a	+	+	*Parvimonas pharyngis* strain G1604, pharynx of coal miners	PQ809993	98%
8b	+	−	-		
9a	+	−	-		
9b	+	+	Uncultured *Parvimonas* sp. clone SM10-68, bovine uterus	JN167637	99%
9c	+	−	-		
10	+	+	Uncultured *Bacteroidetes bacterium* clone P-02, BDD	GQ424167	100%
11	+	−	-		

β-Act: beta-actin (samples were PCR-screened for this house-keeping gene to ascertain their PCR-compatible quality). +: PCR-positive; −: PCR-negative.

**Table 5:** qPCR-positive samples: numerical summary.

Bacterial qPCR Target	Sample Group	Positive Samples	Positive Cows	Range of DNA Copies/2 µL	Median	IQR	U; *p*
*Trueperella pyogenes*	BDD-CHL	18 (100%)	15 (100%)	26–44,512	1785	2704.5	63; 0.33 ^§^
	DDS	17 (100%)	11 (100%)	82–1,866,531	10,608.5	194,876.9	
*Fusobacterium necrophorum*	BDD-CHL	18 (100%)	15 (100%)	624–6,728,847	334,565	1,012,189	87; 0.027 **^§^**
	DDS	17 (100%)	11 (100%)	37–84,815,917	3,272,423	28,358,925.75	
*Treponema pedis*	BDD-CHL	18 (100%)	15 (100%)	1–3,074,616	170,837.5	1,210,763.25	146; 0.0009 ^§^
	DDS	5 (29.4%)	4 (36.3%)	0–876,824	0	44	

Median: 50th percentile; IQR: interquartile range (Q3-Q1); Median, IQR, and statistics were calculated from (mean) DNA loads per cow; ^§^: without Bonferroni correction; Median, IQR, U, and *p* values were calculated from (mean) bacterial DNA loads per cow.

## Data Availability

The original contributions presented in this study are included in the article. Further inquiries can be directed to the corresponding author.

## Supplementary Materials

The following supporting information can be downloaded at: https://www.mdpi.com/article/10.3390/microorganisms14071542/s1, Table S1: Information on BDD-CHL and DDS affected cows. Ref. [69] is inclued in the Supplementary Materials.

## Abbreviations

The following abbreviations are used in this manuscript:

BDD	Bovine digital dermatitis
BDD-CHL	BDD-associated claw horn lesion
DDS	Deep digital sepsis
qPCR	Quantitative PCR
