# Supplementary material for: Do Treponemes in Bovine Digital Dermatitis-Associated Claw Horn Lesions Preclude Co-Infection by Pyogenic Bacteria?
Source: Microorganisms. 2026 Jul 15;14(7):1542. doi: 10.3390/microorganisms14071542 (PMC13414366; doi:10.3390/microorganisms14071542)
Supplement: Supplementary file 1 [file microorganisms-14-01542-s001.zip › microorganisms-4417809-supplementary.pdf]

Supplementary Table S1. Information on BDD-CHL and DDS affected cows

Samples	Information on affected animals (sample source) and pre-treatment before referral to the clinic					
	**Breed**	**Sex**	**Age**	**DIM**	**LCS**	**Housing**	**Pre-treatment**
**BDD-CHL in 15 cows from two different farms in Austria**							
A	SI	F	4	83	3	loose	no
B	SI	F	5	205	4	loose	Block at partner claw, spray
C	SI	F	3	120	3	loose	Block at partner claw, spray
D	SI	F	4	93	3	loose	no
E	SI	F	5	75	4	loose	Block at partner claw, spray
F	SI	F	6.5	73	3	loose	no
G	SI	F	4.5	120	3	loose	no
H	SI	F	3.5	153	4	loose	AB, NSAIDs, block
I	SI	F	3.5	200	4	loose	AB, NSAIDs, block
J	SI	F	4	210	4	loose	Block at partner claw, spray
K	SI	F	5	110	3	loose	no
L	SI	F	6	125	3	loose	no
M	SI	F	5	95	3	loose	no
N	SI	F	7	230	3	loose	no
O	SI	F	2.5	130	3	loose	no
**DDS in 11 cows from 11 different farms**							
1	SI	F	8	95	4	loose	Block at partner claw, spray
2	HF	F	2	155	4	loose	AB, NSAIDs, block
3	SI	F	5.5	40	4	loose	AB, NSAIDs
4	CH	F	4	86	5	loose	AB, NSAIDs, block
5	SI	F	1.5	-	5	loose	AB, NSAIDs, block
6	SI	F	8	180	5	loose	block
7	SI	F	4	126	4	loose	Block at partner claw, spray
8	SI	F	6.5	105	3	loose	no
9	SI	F	2.5	120	5	loose	AB, NSAIDs
10	SI	F	4	85	3	loose	no
11	CH	F	1.5	-	5	loose	AB, NSAIDs

SI: Simmental; HF: Holstein Friesian; CH: Charolais; F: female; Age: in years; DIM: days in milk; LCS: locomotion scoring system (from 1 = normal, to 5 = severely lame) [1]. AB: systemic antibiotic treatment; NSAIDs: nonsteroidal anti-inflammatory drugs; spray: oxytetracycline spray

1. Sprecher DJ, Hostetler DE, Kaneene JB. A lameness scoring system that uses posture and gait to predict dairy cattle reproductive performance. *Theriogenology*. Apr 15 1997;47(6):1179–87. doi:10.1016/s0093-691x(97)00098-8
